# The complete mitochondrial genome of pygmy red-toothed shrew (*Chodsigoa parva)*

**DOI:** 10.1080/23802359.2019.1640092

**Published:** 2019-07-18

**Authors:** Jiao Qing, Huimin Liu, Haixue Wei, Qiong Wang, Shunde Chen

**Affiliations:** College of Life Sciences, Sichuan Normal University, Chengdu, China

**Keywords:** Bayesian phylogenetic tree, Eulipotyphla, mitogenome structure

## Abstract

The study of pygmy red-toothed shrew (*Chodsigoa parva*), which is the smallest species of geuns *Chodsigoa,* is extremely lacking. Also, it is classified as data deficient (DD) on The IUCN Red List. Here, we obtained a complete mitochondrial genome of *Chodsigoa parva*. The mitochondrial genome of *C. parva* is totally 17,216 bp in length and it is composed of 13 protein-coding genes, 22 transfer RNA genes (tRNA), 2 ribosomal RNA genes (rRNA), and 2 non-coding regions. We restructured Bayesian phylogenetic tree by using 19 species those belong to family Soricidae. The mitochondrial genome can provide basic data for further study about the phylogenetic relationship of family Soricidae.

Pygmy red-toothed shrew (*Chodsigoa parva*) belongs to subfamily Soricinae, which is the smallest species of geuns *Chodsigoa* (Chen et al. 2017) and its type locality is Lijiang, Yunnan Province (Allen 1923). Originally, this species is considered as a subspecies of *Chodsigoa hypsibia* by Allen (1923), subsequently Hoffmann (1985) regarded it as a subspecies of *Chodsigoa lamula*, now *C. parva* has been elevated to species status (Lunde et al. 2003). The habitat and distribution of *C. parva* are not yet clear, due to the lack of comprehensive biological data (Hoffmann and Lunde 2008). *C. parva* is classified as data deficient (DD) on The IUCN Red List (www.iucnredlist.org). In this study, we sequenced complete mitochondrial genome of *C. parva* (GenBank Number: MN038167) and restructured Bayesian phylogenetic tree with other 18 shrew species to infer its phylogenetic status.

This individual was captured in Ningwu County, Shanxi Province at an altitude of 1970 m (Latitude: 38.72332°N, Longitude: 111.91465°E) and the specimen of *C. parva* is maintained in Sichuan Academy of Forestry, Chengdu (voucher number: SAF10222).

The whole mitochondrial genome of *C. parva* is 17,216 bp, which contains 13 protein-coding genes, 2 ribosomal RNA genes (rRNA), 22 transfer RNA genes (tRNA), and 2 non-coding regions. The total length of 13 protein-coding genes is 11,418 bp and all of the protein-coding genes begin with ATG, except for *ND2, ND5* (ATA), and *ND3* (ATT). Termination codons of seven protein-coding genes (*COX1, COX2, ATP8, ATP6, ND4L, ND5* and *ND6*) are TAA and termination codons of five protein-coding genes (*ND1, ND2, COX3, ND3* and *ND4*) are incomplete, only Cyt b uses AGA as termination codon. The two non-coding regions are composed of a light strand replication origin (O_L_) and a D-loop region. The light strand replication origin (O_L_) has a length of 41 bp and is located between tRNA^Asn^ and tRNA^Cys^, the D-loop region has a length of 1739 bp and is located between tRNA^Pro^ and tRNA^Phe^.

Thirteen mitochondrial protein genes from *C. parva* and other 18 species were used for phylogenetic analysis. Four species of geuns *Crodura* were used as outgroups. The jModeltest 2.1.3 (Darriba et al. 2012) was used to determine the model of evolution by AIC (Akaike Information Criterion). The best substitution model was GTR. BEAST v1.6.1 (Drummond et al. 2012) was used to restructure Bayesian phylogenetic tree (Figure 1).

**Figure 1.**
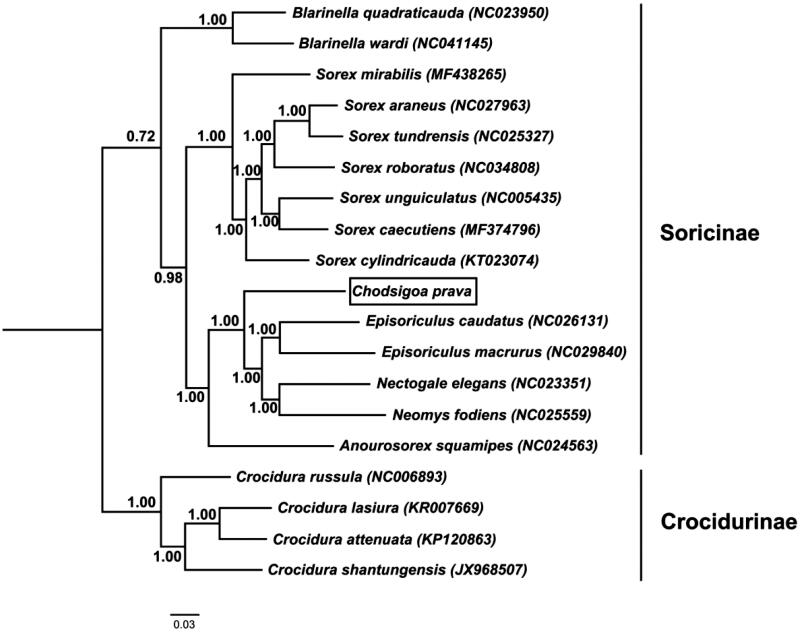
Bayesian phylogenetic tree based on 13 protein genes of mitochondrial genome. Numbers by the nodes indicate Bayesian posterior probabilities.

The Bayesian phylogenetic tree shows that subfamily Soricinae are divided into three groups: geuns *Blarinella*, geuns *Sorex* and the rest genera containing *Chodsigoa*, *Episoriculus*, *Nectogale*, *Neomys* and *Anourosorex*. *C. parva* is located in the basal position of tribe Nectogalini and it is well supported (BPP = 1.00).

Our study contributes to clarify taxonomic status and mitochondrial genome structure of *C. parva*. However, phylogenetic and evolution studies of tribe Nectogalini is still controversial and more data need to be collected for further study.
